# Supplementary material for: Are clinically unimportant findings qualified as benign in lumbar spine imaging reports? A content analysis of plain X-ray, CT and MRI reports
Source: PLoS One. 2024 Mar 13;19(3):e0297911. doi: 10.1371/journal.pone.0297911 (PMC10936854; doi:10.1371/journal.pone.0297911)
Supplement: S1 Table — *Bracketed numbers indicate the studies that determined the findings are likely clinically unimportant. (DOCX) [file pone.0297911.s001.docx]

**S1 Table: Likely clinically unimportant findings based upon the published evidence for the relevance of imaging findings***

| Finding | X-Ray | CT | MRI |
| --- | --- | --- | --- |
| Alignment, malalignment (1, 2) | X | X | X |
| Annular bulge, bulging (1) |  |  | X |
| Annular fissure, fissuring, disruption, tear (1, 3, 4) |  |  | X |
| Anterolisthesis, Grade 1 (1) | X | X | X |
| Baastrup’s syndrome (5) | X |  |  |
| Bone island (6, 7) | X | X |  |
| Bony hypertrophy (8) |  | X | X |
| Cyst, synovial (9) |  |  | X |
| Cyst, Tarlov (10) |  |  | X |
| Degenerative changes or disease (1, 2, 8) | X | X | X |
| Degenerative changes, bone (8) |  |  | X |
| Degenerative changes, intervertebral (1, 8) |  |  | X |
| Disc (gas) vacuum phenomenon or effect, gas present, degenerative gas within (11, 12) |  | X |  |
| Disc (space) height loss/reduction or narrowing, degeneration, width, rudimentary (1, 2, 4, 8) | X | X | X |
| Disc abnormality, changes, contour, morphology, margin (flattening), uncovering (1-3) |  | X | X |
| Disc annulus bulge/bulging (1, 2) |  |  | X |
| Disc bulge, bulging (1, 2, 4) |  | X | X |
| Disc calcified (8, 13) |  | X |  |
| Disc cartilage narrowing (8) | X |  |  |
| Disc degenerative changes or disease, degenerate, degeneration, disruption, discogenic degeneration (1-4) | X | X | X |
| Disc fragment, herniation (2, 3) |  | X | X |
| Disc lesion (2) | X | X | X |
| Disc osteophyte complex (8) |  | X | X |
| Disc prolapse (1) |  | X | X |
| Disc protrusion, protrudes, projects posteriorly (1, 3, 4) | X | X | X |
| Disc signal [loss], hydration, dessication (1, 4) |  |  | X |
| Endplate degenerative (signal) change or oedema, fatty (Modic Type 2) (2) | X |  | X |
| Endplate spurring, lipping, irregularity, pathology, bony spurs, osteophytes, sclerosis (8) | X | X | X |
| Epidural fat, reduced, indentation (2) |  |  | X |
| Epidural space effacement, mild (2) |  |  | X |
| Facet (or apophyseal) joint degeneration, degenerative change or disease (any severity), arthropathy, arthrosis (1, 3, 4) | X | X | X |
| Facet joint effusions, joint fluid, oedema (adjacent), synovitis, joint cyst due to OA (1) |  |  | X |
| Facet joint osteoarthritis, arthritic changes, hypertrophy, bony irregularity (1, 3) | X | X | X |
| Focal fat rest(14) |  |  | X |
| Foraminal stenosis, narrowing, encroachment (mild) (4) |  | X | X |
| Gas, intra-articular (11, 12) |  | X |  |
| Granuloma, injection calcified(15) | X |  |  |
| Haemangioma(16) |  | X | X |
| Haematoma, calcified(15) |  |  | X |
| Joint space loss, narrowing(1, 2, 8) |  | X |  |
| Kyphosis, kyphotic curvature (mild)(17) | X | X |  |
| Lesion, calcified soft tissue(15) | X |  |  |
| Ligament, facet (thickened)(1) |  |  | X |
| Ligamentum flavum (flava ligament), hypertrophy, thickening (2) |  | X | X |
| Lumbar lordosis, straightened/normal, increased(18) | X | X | X |
| Lumbar-type vertebrae, lumbosacral transitional anatomy, sacralisation of transverse processes, sacralised, Pseudoarthrosis, lumbarisation, hemisacralisation, elongated transverse processes (19, 20) | X | X | X |
| Modic changes, Type 2 or 3 (2) |  |  | X |
| Multilevel pathology (1, 2, 8) |  | X |  |
| Nerve root contact or displacement (without compression), mild indentation (4) |  | X | X |
| Osteoarthritis (1, 2, 8) |  |  | X |
| Osteophytes, osteophytic change, spurring, lipping, marginal/endplate, osteophytosis (8) | X | X | X |
| Pars defect (21) | X | X | X |
| Pedicles, congenitally short, foreshortening(22) | X | X | X |
| Pelvic tilt(23) | X |  |  |
| Perineural fat loss(4, 24) |  |  | X |
| Radicular irritation (4) |  |  | X |
| Retrolisthesis, posterior slip/listhesis (mild)(25) | X | X | X |
| Sacroiliac (SI) joint, narrowing, degenerative change, arthropathy, osteoarthritic changes, partial fusion, marginal signal change, joint fluid (26, 27) | X | X | X |
| Schmorl's nodes (2) |  | X | X |
| Scoliosis, curvature, curve, straightening (minor/mild, in adults)(28, 29) | X | X | X |
| Spinous processes, contact, sclerosis (5) | X | X |  |
| Spondylolisthesis, listhesis or anterior slip or displacement, Grade 1, degenerative (1, 2, 4) | X | X | X |
| Spondylolysis (21) | X |  | X |
| Spondylosis, spondylitic changes/lipping (8) | X | X | X |
| Stenosis or compromise, lateral recess (mild), congenital (4, 30) |  | X | X |
| Stenosis, narrowing or encroachment, central canal (mild), focal features, developmental, acquired (2, 4, 31) |  | X | X |
| Syndesmophytes (8) | X |  |  |
| Thecal sac indentation, bulging, encroachment, displacement, compression, impingement, effacement (mild) (2) |  | X | X |
| Vertebral body degenerative features (at margins) (8) |  |  | X |
| Vertebral body height loss, wedging <15 - 20% (31, 32) |  |  | X |

*Bracketed numbers indicate the studies that determined the findings are likely clinically unimportant.

**References**

1. Brinjikji W, Luetmer PH, Comstock B, Bresnahan BW, Chen LE, Deyo RA, et al. Systematic literature review of imaging features of spinal degeneration in asymptomatic populations. AJNR Am J Neuroradiol. 2015;36(4):811-6.
2. Kasch R, Truthmann J, Hancock MJ, Maher CG, Otto M, Nell C, et al. Association of Lumbar MRI Findings with Current and Future Back Pain in a Population-based Cohort Study. Spine. 2022;47(3):201-11.
3. Smith A, Hancock M, O'Hanlon S, Krieser M, O'Sullivan P, Cicuttini F, et al. The Association Between Different Trajectories of Low Back Pain and Degenerative Imaging Findings in Young Adult Participants Within The Raine Study. Spine (Phila Pa 1976). 2022;47(3):269-76.
4. Jarvik JG, Comstock BA, James KT, Avins AL, Bresnahan BW, Deyo RA, et al. Lumbar Imaging With Reporting Of Epidemiology (LIRE)--Protocol for a pragmatic cluster randomized trial. Contemp Clin Trials. 2015;45(Pt B):157-63.
5. Kwong Y, Rao N, Latief K. MDCT Findings in Baastrup Disease: Disease or Normal Feature of the Aging Spine? American Journal of Roentgenology. 2011;196(5):1156-9.
6. Greenspan A. Bone island (enostosis): current concept--a review. Skeletal Radiol. 1995;24(2):111-5.
7. Palmer W, Bancroft L, Bonar F, Choi JA, Cotten A, Griffith JF, et al. Glossary of terms for musculoskeletal radiology. Skeletal Radiol. 2020;49(Suppl 1):1-33.
8. Chen L, Perera RS, Radojcic MR, Beckenkamp PR, Ferreira PH, Hart DJ, et al. Association of Lumbar Spine Radiographic Changes With Severity of Back Pain-Related Disability Among Middle-aged, Community-Dwelling Women. JAMA Netw Open. 2021;4(5):e2110715.
9. Khan AM, Girardi F. Spinal lumbar synovial cysts. Diagnosis and management challenge. European Spine Journal. 2006;15(8):1176-82.
10. Paulsen RD, Call GA, Murtagh FR. Prevalence and percutaneous drainage of cysts of the sacral nerve root sheath (Tarlov cysts). American Journal of Neuroradiology. 1994;15(2):293-7.
11. Feng S-W, Chang M-C, Wu H-T, Yu J-K, Wang S-T, Liu C-L. Are intravertebral vacuum phenomena benign lesions? European Spine Journal. 2011;20(8):1341-8.
12. D’Anastasi M, Birkenmaier C, Schmidt GP, Wegener B, Reiser MF, Baur-Melnyk A. Correlation Between Vacuum Phenomenon on CT and Fluid on MRI in Degenerative Disks. American Journal of Roentgenology. 2011;197(5):1182-9.
13. Chanchairujira K, Chung CB, Kim JY, Papakonstantinou O, Lee MH, Clopton P, et al. Intervertebral Disk Calcification of the Spine in an Elderly Population: Radiographic Prevalence, Location, and Distribution and Correlation with Spinal Degeneration. Radiology. 2004;230(2):499-503.
14. ande Berg BC, Malghem J, Lecouvet FE, Maldague B. Magnetic resonance imaging of the normal bone marrow. Skeletal Radiology. 1998;27(9):471-83.
15. Freire V, Moser TP, Lepage-Saucier M. Radiological identification and analysis of soft tissue musculoskeletal calcifications. Insights Imaging. 2018;9(4):477-92.
16. Peckham ME, Hutchins TA. Imaging of Vascular Disorders of the Spine. Radiol Clin North Am. 2019;57(2):307-18.
17. Fon GT, Pitt MJ, Thies AC. Thoracic kyphosis: range in normal subjects. American Journal of Roentgenology. 1980;134(5):979-83.
18. Been E, Kalichman L. Lumbar lordosis. Spine J. 2014;14(1):87-97.
19. Bron JL, van Royen BJ, Wuisman PI. The clinical significance of lumbosacral transitional anomalies. Acta orthopaedica Belgica. 2007;73(6):687-95.
20. Otani K, Konno S, Kikuchi S. Lumbosacral transitional vertebrae and nerve-root symptoms. The Journal of Bone and Joint Surgery British volume. 2001;83-B(8):1137-40.
21. yrmou E, Tsitsopoulos PP, Marinopoulos D, Tsonidis C, Anagnostopoulos I, Tsitsopoulos PD. Spondylolysis: a review and reappraisal. Hippokratia. 2010;14(1):17-21.
22. Soldatos T, Chalian M, Thawait S, Belzberg AJ, Eng J, Carrino JA, et al. Spectrum of magnetic resonance imaging findings in congenital lumbar spinal stenosis. World journal of clinical cases. 2014;2(12):883-7.
23. Le Huec JC, Aunoble S, Philippe L, Nicolas P. Pelvic parameters: origin and significance. Eur Spine J. 2011;20 Suppl 5(Suppl 5):564-71.
24. Lee S, Lee JW, Yeom JS, Kim K-J, Kim H-J, Chung SK, et al. A Practical MRI Grading System for Lumbar Foraminal Stenosis. American Journal of Roentgenology. 2010;194(4):1095-8.
25. 2hen M, Razi A, Lurie JD, Hanscom B, Weinstein J. Retrolisthesis and lumbar disc herniation: a preoperative assessment of patient function. The Spine Journal. 2007;7(4):406-13.
26. YAGAN R, KHAN MA, MARMOLYA G. Role of Abdominal CT, When Available in Patients' Records, in the Evaluation of Degenerative Changes of the Sacroiliac Joints. Spine. 1987;12(10):1046-51.
27. Asada M, Tokunaga D, Arai Y, Oda R, Fujiwara H, Yamada K, et al. Degeneration of the Sacroiliac Joint in Hip Osteoarthritis Patients: A Three-Dimensional Image Analysis. Journal of the Belgian Society of Radiology. 2019;103(1):36.
28. chwab F, Dubey A, Gamez L, El Fegoun AB, Hwang K, Pagala M, et al. Adult scoliosis: prevalence, SF-36, and nutritional parameters in an elderly volunteer population. Spine (Phila Pa 1976). 2005;30(9):1082-5.
29. Van Goethem J, Van Campenhout A, van den Hauwe L, Parizel PM. Scoliosis. Neuroimaging Clin N Am. 2007;17(1):105-15.
30. Splettstößer A, Khan MF, Zimmermann B, Vogl TJ, Ackermann H, Middendorp M, et al. Correlation of lumbar lateral recess stenosis in magnetic resonance imaging and clinical symptoms. World journal of radiology. 2017;9(5):223-9.
31. Traeger AC, Underwood M, Ivers R, Buchbinder R. Low back pain in people aged 60 years and over. BMJ. 2022;376:e066928.
32. 3Vogt TM, Ross PD, Palermo L, Musliner T, Genant HK, Black D, et al. Vertebral Fracture Prevalence Among Women Screened for the Fracture Intervention Trial and a Simple Clinical Tool to Screen for Undiagnosed Vertebral Fractures. Mayo Clinic Proceedings. 2000;75(9):888-96.
