# Supplementary material for: Are clinically unimportant findings qualified as benign in lumbar spine imaging reports? A content analysis of plain X-ray, CT and MRI reports
Source: PLoS One. 2024 Mar 13;19(3):e0297911. doi: 10.1371/journal.pone.0297911 (PMC10936854; doi:10.1371/journal.pone.0297911)
Supplement: S2 Table — (DOCX) [file pone.0297911.s002.docx]

**S2 Table: Clinically unimportant findings that were reported to exclude the presence of the pathology by number of reports and overall frequency of the finding**

|  | **Number of reports where finding is reported to be absent, N (% of reports)** | **Frequency of absent finding across all reports, N (% of findings)** |
| --- | --- | --- |
| **X-ray** | **N = 80 reports** | **N=19 findings** |
| Pars defect | 6 (8) | 6 (32) |
| Facet joint arthropathy | 4 (5) | 4 (21) |
| Degenerative disc disease | 2 (3) | 2 (11) |
| Disc height loss | 2 (3) | 2 (11) |
| Disc (general) | 1 (1) | 1 (5) |
| Degeneration (general) | 1 (1) | 1 (5) |
| Endplate pathology | 1 (1) | 1 (5) |
| Pelvic tilt | 1 (1) | 1 (5) |
| Sacro-iliac joint degeneration | 1 (1) | 1 (5) |
| **CT** | **N=82 reports** | **N=82 findings** |
| Facet joint arthropathy | 14 (17) | 16 (20) |
| Disc protrusion | 12 (15) | 15 (18) |
| Pars defect | 13 (16) | 13 (16) |
| Disc herniation | 10 (12) | 11 (13) |
| Disc (general) | 6 (7) | 8 (10) |
| Disc prolapse | 5 (6) | 6 (7) |
| Disc height loss | 3 (4) | 3 (4) |
| Sacro-iliac joint degeneration | 3 (4) | 3 (4) |
| Disc bulge | 2 (2) | 2 (2) |
| Alignment | 1 (1) | 1 (1) |
| Degeneration (general) | 1 (1) | 1 (1) |
| Lordosis | 1 (1) | 1 (1) |
| Spinous process | 1 (1) | 1 (1) |
| Nerve root contact | 1 (1) | 1 (1) |
| **MRI** | **N=100 reports** | **N=107 findings** |
| Disc protrusion | 21 (21) | 30 (28) |
| Disc bulge | 17 (17) | 20 (19) |
| Pars defect | 19 (19) | 19 (18) |
| Facet joint arthropathy | 13 (13) | 16 (15) |
| Disc herniation | 5 (5) | 5 (5) |
| Disc (general) | 4 (4) | 4 (4) |
| Disc prolapse | 3 (3) | 4 (4) |
| Lumbar spine alignment | 2 (2) | 2 (2) |
| Disc height loss | 2 (2) | 2 (2) |
| Annular fissure | 1 (1) | 1 (1) |
| General degeneration | 1 (1) | 1 (1) |
| Sacro-iliac joint degeneration | 1 (1) | 1 (1) |
| Syndesmophytes | 1 (1) | 1 (1) |
| Tarlov cyst | 1 (1) | 1 (1) |
