# Supplementary material for: Are clinically unimportant findings qualified as benign in lumbar spine imaging reports? A content analysis of plain X-ray, CT and MRI reports
Source: PLoS One. 2024 Mar 13;19(3):e0297911. doi: 10.1371/journal.pone.0297911 (PMC10936854; doi:10.1371/journal.pone.0297911)
Supplement: S3 Table — ^N = number of reports (% of all reports for same modality). #N = number of times finding present (% of all findings for same modality). αN = number of times finding reported as present (% of same finding). (DOCX) [file pone.0297911.s003.docx]

**S3 Table: Frequency and type of likely clinically important findings by modality**

|  | **Number of reports where finding is reported** | **Frequency of finding across reports** | **Number of findings reported to be present** |
| --- | --- | --- | --- |
| **X-ray** | **N=80 reports**  **N (%)^^^** | **N=179 findings**  **N (%)^#^** | **N=57 findings**  **N (%)^α^** |
| Fracture | 52 (65) | 61 (34) | 2 (3) |
| Suspicious lesion | 21 (26) | 22 (12) | 0 (0) |
| Surgical changes | 9 (11) | 20 (11) | 20 (100) |
| Scoliosis | 15 (19) | 18 (10) | 17 (94) |
| Spondylolisthesis | 12 (15) | 13 (7) | 3 (23) |
| Sacro-iliac joint pathology | 4 (5) | 9 (5) | 2 (22) |
| Bony morphology | 7 (9) | 8 (4) | 2 (25) |
| Bone erosion | 7 (9) | 7 (4) | 0 (0) |
| Osteoporosis | 5 (6) | 5 (3) | 3 (60) |
| Soft tissue | 5 (6) | 5 (3) | 1 (20) |
| Abnormality (general) | 4 (5) | 4 (2) | 0 (0) |
| Sclerosis | 3 (4) | 3 (2) | 3 (100) |
| Instability | 2 (3) | 2 (1) | 0 (0) |
| Retrolisthesis | 1 (1) | 2 (1) | 2 (100) |
| **CT** | **N=82 reports**  **N (%)^^^** | **N=466 findings**  **N (%)^#^** | **N=97 findings**  **N (%)^α^** |
| Canal stenosis | 61 (74) | 121 (26) | 32 (26) |
| Foraminal stenosis | 48 (59) | 88 (19) | 23 (26) |
| Fracture | 56 (68) | 73 (16) | 0 (0) |
| Nerve root compression | 25 (30) | 46 (10) | 17 (37) |
| Suspicious lesion | 24 (29) | 26 (6) | 0 (0) |
| Soft tissue pathology | 21 (26) | 26 (6) | 0 (0) |
| Spondylolisthesis | 14 (17) | 14 (3) | 1 (7) |
| Surgical changes | 3 (4) | 11 (2) | 10 (91) |
| Bone erosion | 10 (12) | 10 (2) | 0 (0) |
| Lateral recess | 7 (9) | 9 (2) | 6 (67) |
| Cancer | 7 (9) | 7 (2) | 1 (14) |
| Abnormality (general) | 6 (7) | 6 (1) | 0 (0) |
| Extrusion | 4 (5) | 4 (1) | 0 (0) |
| Osteoporosis | 4 (5) | 4 (1) | 3 (75) |
| Scoliosis | 4 (5) | 4 (1) | 3 (75) |
| Infection | 2 (2) | 3 (1) | 0 (0) |
| Mass | 2 (2) | 3 (1) | 0 (0) |
| Bone morphology | 3 (4) | 3 (1) | 0 (0) |
| Thecal compression | 3 (4) | 3 (1) | 1 (33) |
| Epidural collection or mass | 1 (1) | 2 (<1) | 0 (0) |
| Conus or cord pathology | 1 (1) | 1 (<1) | 0 (0) |
| Inflammation | 1 (1) | 1 (<1) | 0 (0) |
| Marrow | 1 (1) | 1 (<1) | 0 (0) |
| **MRI** | **N=100 reports**  **N (%)^^^** | **N=871 findings**  **N (%)^#^** | **N=201 findings**  **N (%)^α^** |
| Foraminal stenosis | 71 (71) | 153 (18) | 25 (16) |
| Canal stenosis | 68 (68) | 146 (17) | 27 (18) |
| Nerve root impingement | 74 (74) | 134 (15) | 50 (37) |
| Conus and cord pathology | 75 (75) | 88 (10) | 1 (1) |
| Fracture | 50 (50) | 66 (8) | 6 (9) |
| Marrow abnormalities | 42 (42) | 56 (6) | 11 (20) |
| Soft tissue abnormalities | 36 (36) | 42 (5) | 3 (7) |
| Disc extrusion | 25 (25) | 32 (4) | 22 (69) |
| Surgical changes | 10 (10) | 19 (2) | 16 (84) |
| Lateral recess stenosis | 15 (15) | 17 (2) | 9 (53) |
| Abnormality (general) | 14 (14) | 16 (2) | 1 (6) |
| Spondylolisthesis | 14 (14) | 15 (2) | 2 (13) |
| Cauda equina pathology | 11 (11) | 11 (1) | 0 (0) |
| Suspicious lesion | 8 (8) | 9 (1) | 0 (0) |
| MRI signal changes | 8 (8) | 8 (1) | 6 (75) |
| Arachnoiditis | 7 (7) | 7 (1) | 0 (0) |
| Retrolisthesis | 6 (6) | 7 (1) | 0 (0) |
| Thecal compression | 7 (7) | 7 (1) | 4 (57) |
| Modic 1 changes | 6 (6) | 6 (1) | 6 (100) |
| Inflammation | 5 (5) | 5 (1) | 1 (20) |
| Other nerve pathology | 3 (3) | 5 (1) | 3 (60) |
| Bone erosion | 4 (4) | 4 (<1) | 1 (25) |
| Sacroiliac joint pathology | 4 (4) | 4 (<1) | 1 (25) |
| Epidural collection or mass | 3 (3) | 3 (<1) | 0 (0) |
| Facet joint pathology | 2 (2) | 2 (<1) | 2 (100) |
| Scoliosis | 2 (2) | 2 (<1) | 0 (0) |
| Subluxation | 2 (2) | 2 (<1) | 0 (0) |
| Demyelination | 1 (1) | 1 (<1) | 0 (0) |
| Infection | 1 (1) | 1 (<1) | 1 (100) |
| Instability | 1 (1) | 1 (<1) | 1 (100) |
| Schwannoma | 1 (1) | 1 (<1) | 1 (100) |
| Sclerosis | 1 (1) | 1 (<1) | 1 (100) |

^^^N=number of reports (% of all reports for same modality)

^#^N=number of times finding present (% of all findings for same modality)

^α^N=number of times finding reported as present (% of same finding)
